# The impact of electromagnetic fields generated by high-voltage power lines on the spatial arrangement of pike (*Esox Lucius* Linnaeus 1758) embryos

**DOI:** 10.1007/s11356-024-34300-y

**Published:** 2024-07-12

**Authors:** Jan Krzystolik, Adam Tański, Radosław Piesiewicz, Krzysztof Formicki

**Affiliations:** Department of Hydrobiology, Ichthyology and Biotechnology of Animal Reproduction, West Pomeranian University of Technology, Szczecin, Poland

**Keywords:** Electromagnetic fields, Anthropogenic impact, Fish embryogenesis, Freshwater fish, Fish eggs, Lethal factor

## Abstract

Urbanization and technological advancements result in the dispersion of antropogenic electromagnetic fields (EMF) that can affect on ecosystems. Therefore, it is important to understand their impact on the environment. Aquatic ecosystems are subject to EMF as part of various electricity sources, e.g., high-voltage transmission lines (HVTL). We examined the impact of EMF generated by HVTL on the spatial arrangement and survival of pike (*Esox lucius*) embryos. Fertilized eggs were incubated under two HVTL configurations 110 kV and 220 kV compared with a control group devoid of anthropogenic EMF. Embryo orientation and survival were monitored until blastopore closure. The control group showed dominance in the arrangement of embryos along the N-S, NNW-SSE, and NNE-SSW axes, with a slight prevalence of northern directions. EMF originating from HVTL did not exert a significant influence on the spatial arrangement of pike embryos, although some deviations from the arrangement noticed in the control group were observed. Increased embryo mortality was observed only at 110 kV site, but probably due to factors unrelated to EMF. In conclusion, EMF generated by HVTL did not significantly change pike embryo orientation or chances of survival. However, longer exposure or higher EMF levels could provoke notable reactions, requiring ongoing evaluation as power networks continue to spread more widely.

## Introduction

With the dynamic advancement of technology and the ongoing urbanization of our societies, human-generated electromagnetic fields (EMF) have become an integral aspect of daily life. The escalating demand for electrical energy each year (Chakraborty et al. 2023) leads to a rise in the number of objects generating electromagnetic fields, including all entities involved in producing, transmitting, or utilizing electrical energy. Although the impact of EMF on biological structures was confirmed nearly half a century ago (Marino and Becker 1977), the continual increase in the number of EMF sources in recent years has prompted growing interest in their influence on ecosystems and specific groups of living organisms (Brizhik 2011; Cucurachi et al. 2013; Vasilyeva et al. 2021; Levitt et al. 2022).

Aquatic ecosystems are also subjected to expanding influences of EMF, with offshore wind farms and underwater power cables frequently identified as primary sources (Andrulewicz et al. 2003; Öhman et al. 2007; Gill et al. 2012; Fey et al. 2019a). Another source of electromagnetic fields may be high-voltage transmission lines (HVTL). Due to the density of HVTL in both Poland and most European countries, they represent one of the most prevalent sources of EMF affecting inland water ecosystems. The values of EMF generated by HVTL depend on factors such as current intensity, electrical voltage, and line load, making them variable over time (Olsen and Wong 1992; Chen et al. 2022). Another crucial factor in considering the impact of EMF from high-voltage lines is the distance from the lines, as field values decrease with increasing distance (Al-Bassam et al. 2016).

Considering the factors mentioned above, EMF from HVTL can generally be characterized as low-frequency and having relatively low values (Olsen and Wong 1992). This raises the question of whether even fields with low values, such as those generated by HVTL, can impact aquatic ecosystems.

Research addressing the impact of EMF generated by underwater HVTL on aquatic organisms shows a noticeable, though often minor, influence on their behaviors (Andrulewicz et al. 2003; Gill et al. 2012; Scott et al. 2021). There are indications that EMF generated by underwater cables may be detectable and, in some cases, affect the migration of anadromous fish species such as Chinook salmon (*Oncorhynchus tshawytscha*), green sturgeon (*Acipenser medirostris*) (Wyman et al. 2018, 2023), European eel (*Anguila anguila*) (Öhman et al. 2007), and rainbow trout (*Oncorhynchus mykiss*) (Jakubowska et al. 2021). However, it is believed that the actual effects of EMF on the migration of anadromous fish are currently relatively minor (Gillson et al. 2022).

Studies conducted on non-anadromous freshwater species, including fathead minnow (*Pimephales promelas*), redear sunfish (*Lepomis microlophus*), striped bass (*Morone saxatilis*), lake sturgeon (*Acipenser fulvescens*), and channel catfish (*Ictalurus punctatus*), yield ambiguous results, indicating that some of the examined species exhibit a behavioral response to the presence of EMF, while others remain indifferent (Bevelhimer et al. 2013).

As EMF can also affect areas crucial for fish reproduction, it is necessary to assess their impact on embryonic development. Previous studies on the influence of a static magnetic field (SMF) and EMF show that these fields can affect various aspects of embryogenesis, including the time and pace of embryo development (Chebotareva et al. 2009; Brysiewicz et al. 2017), heart function (Formicki and Winnicki 1996; Winnicki et al. 2004), permeability of egg membranes (Sadowski et al. 2007), the timing and quantity of melanophores (Testorf et al. 2002; Brysiewicz et al. 2017), mortality (Krylov et al. 2016a), and metabolic rate. With an increase in metabolic rate, a faster resorption of the yolk sac is observed (Fey et al. 2019b), along with a greater mass and length of the incubated hatch under the influence of SMF (Formicki and Winnicki 1998). One aspect of embryogenesis influenced by SMF, among others, is the change in the arrangement of embryo bodies inside the incubating eggs (Formicki et al. 1997; Formicki and Winnicki 1998; Tanski et al. 2005). However, there is a lack of studies demonstrating a clear impact of EMF on this process.

As it is assumed that EMF, even of low frequency and values, exert a significantly stronger influence on biological structures than SMF (Formicki et al. 2021), we decided to investigate whether EMF generated by HVTL would affect the spatial arrangement of pike (*Esox lucius*) embryos. This species was selected because its spawning and subsequent incubation of eggs occur in the littoral waters or even periodically inundated meadows (Zrazomska and Korzyner 1958; Casselman 1996), thus in shallow waters where the expected impact of EMF generated by HVTL is greater compared to deeper waters.

## Material and methods

### Location of research sites

**Fig. 1:**
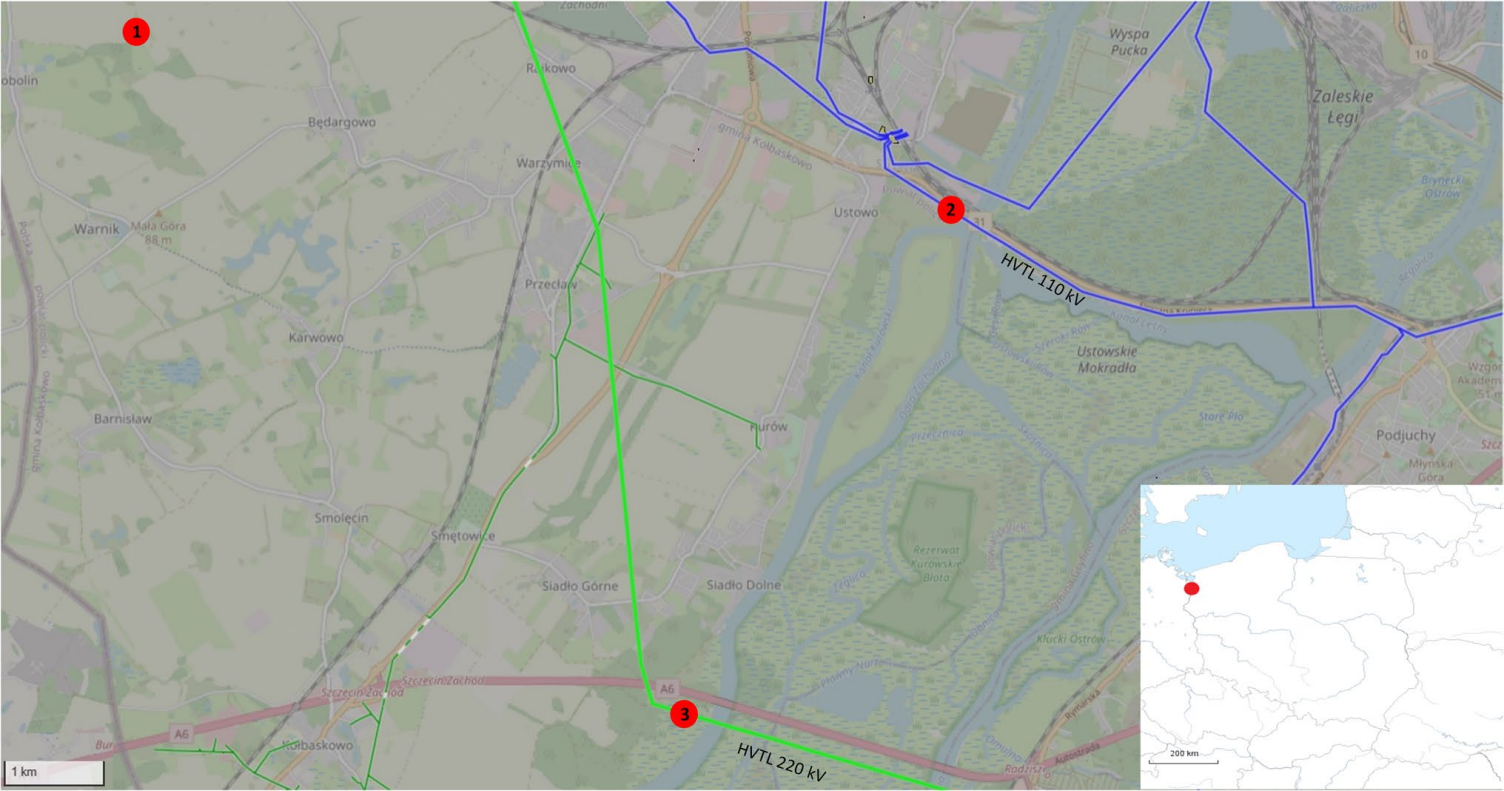
Location of research sites on the map: 1, control point; 2, first research site (110 kV HVTL with a frequency of 50 Hz and EMF value from 0.015 to 0.134 μT); 3, second research site (220 kV HVTL with a frequency of 50 Hz and EMF value from 0.523 to 0.956 μT) (based on the OpenStreetMap database)

The experiment location (Fig. 1) was selected to have high-voltage transmission lines (HVTL) directly near the Oder River and potential spawning grounds of pike, aiming to achieve the most faithful representation of the real impact of EMF on incubating eggs in a natural environment.

The first research site was set within the city of Szczecin (Poland) on the western bank of the West Oder (53°22′58.379″ N, 14°32′1.661″ E) under 110 kV HVTL with a frequency of 50 Hz. This line runs through the West Oder, along the Forest Canal, and then through the East Oder, potentially exerting a significant impact on aquatic ecosystems. The measured EMF value at this site ranged from 0.015 to 0.134 μT, depending on the measurement time.

The second research site was set on the western bank of the West Oder above the city of Szczecin (Poland) near the A6 motorway (53°20′18.539″ N, 14°29′29.734″ E) under 220 kV HVTL with a frequency of 50 Hz. This line runs through the West Oder, and then crosses the channels of Międzyodrze and the East Oder. The research site was established near the West Oder channel, in the floodplains, which are a significant area for pike spawning. The measured EMF value at this site ranged from 0.523 to 0.956 μT, depending on the measurement time.

In the control sample, the eggs were incubated only in the natural geomagnetic field (GMF). The control sample location was in the fields near the village of Bobolin (Poland), situated at least 1000 m away from any buildings or high-voltage lines to eliminate any anthropogenic EMF (53°24′9.791″ N, 14°24′54.376″ E).

The experiments were conducted in a manner ensuring that high-voltage transmission lines (HVTL) were directly near the Oder River and potential spawning grounds of pike, aiming to achieve the most faithful representation of the real impact of EMF on incubating eggs in a natural environment.

### Material

The material for the research consisted of the fertilized pike eggs. Spawning fish were caught from the waters of the West Oder using trap tools. This method of capture is considered relatively safe for fish and allows their release in good condition after spawning (Czarkowski and Kapusta 2016; Zakęs et al. 2018). Eggs from eight females were collected using the air stripping method, ensuring high-quality gametes (Cejko et al. 2016), while milt from five males was obtained by hand stripping. The eggs were fertilized and then carefully separated. A total of 100 randomly selected egg grains were transported to the laboratory to assess fertilization efficiency. Fertilization assessments were made at the gastrulation stage using microscopic observation.

### Experiment setup

The remaining fertilized eggs were placed in 0.75 dm^3^ thermoses, ensuring a constant temperature during transport (7 ± 1 °C). Portable sets were used for incubating eggs at selected research sites, consisting of oval containers with a volume of 200 L designed for storing food, with light-impermeable walls. Inside the containers, glass columns were placed, with three round reference arenas with a volume of 3 dm^3^ each, on which grids with mesh sides adjusted to the size of the eggs were stretched, ensuring that each egg remained immobile during incubation. In each reference arena, 300 pike eggs were placed, totaling 900 eggs for each research site. The large volume of water guaranteed thermal stability and provided adequate oxygen demand in the initial phase of embryonic development. Additionally, each set was covered with a layer of thermal insulation to shield it from sunlight and prevent temperature drops at night inside the set.

### Result reading and analysis

The positioning of developing embryos was read after the closure of the blastopore, i.e., when the embryo’s body is visible but still not moving. In the case of pike, this usually occurs around 30 degree-days (°D) (Bonisławska et al. 2011). The water temperature in all incubators was continuously monitored to capture the appropriate moment, and individual developing eggs were carefully removed and observed under a microscope to assess the advancement of embryogenesis. This allowed determining the moment when the blastopore closed in the properly developing eggs, which occurred at 37°D of incubation, i.e., after 6 days. Reading at a later stage of embryonic development, when mobility appears, could distort the results. The reading was done after lowering the water level in the incubator and adding acetic acid dropwise to the water in the „reference arenas.” This caused the denaturation of proteins and the visualization of the embryo’s body outline, facilitating easier observation of its positioning. The orientation of the embryos concerning the cardinal directions was determined on-site using a wind rose. Circular statistics were used to determine the direction and plane of pike embryo positioning. The results were subjected to statistical analysis using the Rayleigh test (Batschelet 1981).

In addition, at each research site, the number of dead eggs was counted, and the survival rate until the closure of the blastopore was assessed based on the formula

$$SR=\frac{SE}{n\times FR}\times 100\text{\%}$$

where.

*SR*: is the survival rate;
*SE*: is the number of embryos that survived until blastopore closure;
*n*: is the initial number of eggs (*n* = 900);
*FR*: is the fertilization rate.

The survival results were subjected to analysis using the chi-square independence test.

## Results

The fertilization efficiency of the eggs was 67%, resulting in 603 fertilized eggs in each experimental variant, given the initial number of eggs at each research site (900). The survival of fertilized eggs until the closure of the blastopore was 66.2% in the absence of additional EMF sources (control group), 60.2% under the high-voltage transmission line (HVTL) with 110 kV (chi-square test *χ*^2^ = 4.62; *p* = 0.05), and 68.0% under the HVTL with 220 kV (chi-square test *χ*^2^ = 0.45; *p* = 0.05).

**Fig. 2:**
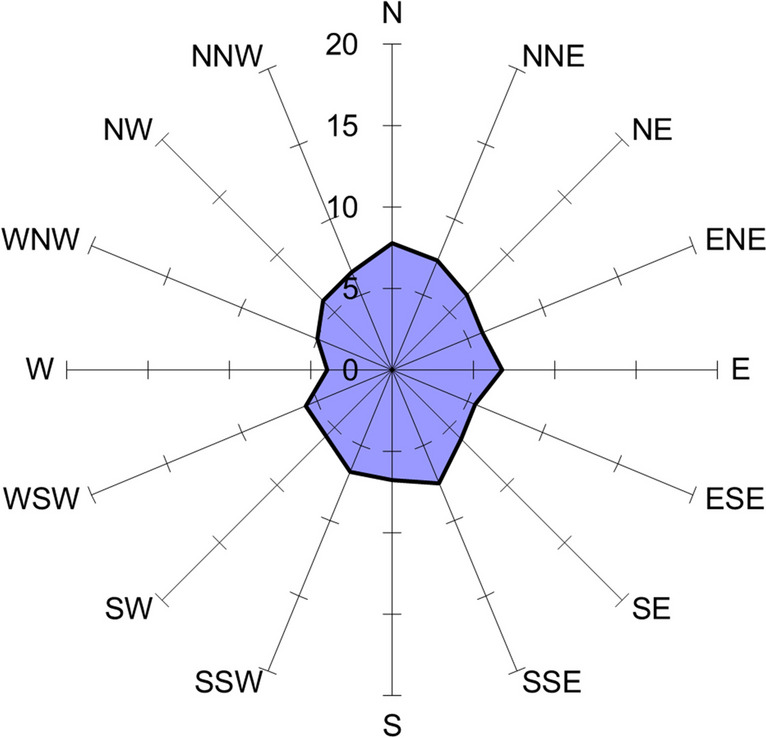
Percentage contribution to the spatial arrangement of pike embryos—control group

Pike embryos incubating without magnetic field disturbances (control group) showed dominance in the arrangement along the N-S, NNW-SSE, and NNE-SSW axes, with a slight prevalence of northern directions. A significant proportion of embryos were also positioned in the W-E plane, with a predominance of the eastern direction (Fig. 2). Nevertheless, there was no statistically significant dominance in any of the directions or axes of embryo arrangement (Rayleigh test *r* = 0.0792, *p* = 0.082).

**Fig. 3:**
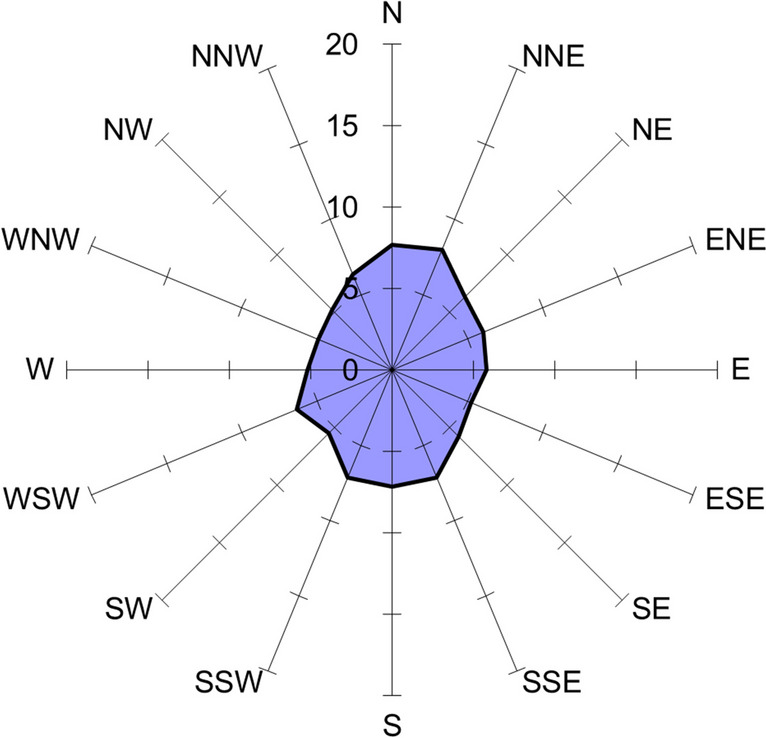
Percentage contribution to the spatial arrangement of pike embryos—high-voltage transmission line 110 kV

At the first research site, where eggs were incubated under the influence of EMF from the 110 kV HVTL, embryo bodies arranged in a similar manner to the control group, with the highest participation along the NNE-SSW, N-S axes (Fig. 3). In this case as well, there was no statistically significant dominance in any of the directions (Rayleigh test *r* = 0.0888, *p* = 0.057).

**Fig. 4:**
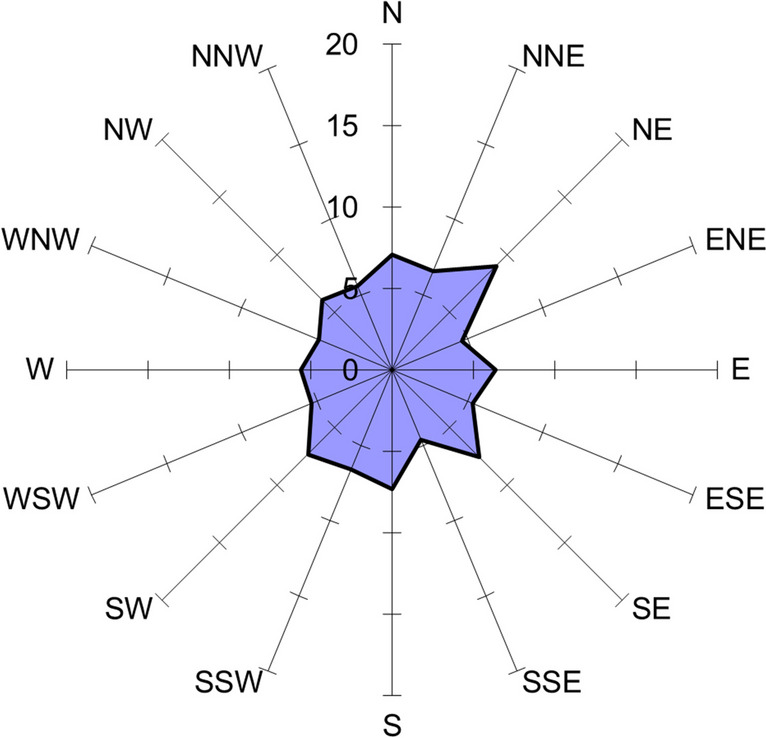
Percentage contribution to the spatial arrangement of pike embryos—high-voltage transmission line 220 kV

There was a change in the dominant axes in the arrangement of embryos in the sample incubated under the 220 kV HVTL (Fig. 4), where the axis NE-SW and the perpendicular axis NW–SE had the highest share, and in these axes, eastern directions predominated (Rayleigh test *r* = 0.0655, *p* = 0.172).

## Discussion

Our observations of the pike eggs in the natural GMF did not show statistically significant dominance in any direction in the arrangement of embryos. However, they indicated a tendency for embryos to align in the N-S, NNW-SSE, and NNE-SSW axes, with a slight dominance of the N direction. These results correspond to earlier research findings (Tanski et al. 2005) on pike and other freshwater fish species, where a preference for embryo alignment in north–south planes (N-S, NNW-SSE, and NNE-SSW) was also noted. It is noteworthy that under the influence of GMF alone, adult individuals of carp (*Cyprinus carpio*) and zebrafish (*Danio rerio*) already showed a clear tendency to align in north–south planes (Hart et al. 2012; Krylov et al. 2016b). This raises questions about the mechanism determining directional alignment in GMF and whether it differs between developing embryos and adult individuals.

The experimental results did not indicate a statistically significant impact of the tested EMF on the directional alignment of pike embryos. Incubating eggs under the 110 kV HVTL induced almost no changes compared to the control group. In this case, embryos also showed a noticeable tendency to align in the NNE-SSW and N-S axes. Greater changes were observed in embryos incubated under the 220 kV HVTL. At this research site, there was a change in orientation, and the majority of embryos aligned in the NE-SW axis. The research results indicate that the EMF generated by the HVTL did not significantly affect the spatial orientation of pike embryos. However, this does not rule out the sensitivity of embryos of this species to magnetic fields, as previous studies have shown that SMF ranging from 0.5 to 4 mT induced a directional reaction in developing pike embryos (Tanski et al. 2005). This may suggest that the EMF generated by the examined HVTL had values too low to evoke a clear directional response. Supporting this notion is the fact that a significant portion of previous studies indicating a significant influence of EMF and SMF on various aspects of embryogenesis was conducted in fields with higher values than those examined in this experiment (Formicki et al. 2021).

Another factor that may contribute to the limited impact of HVTL EMF is the relatively short exposure time to EMF. The experiment lasted only 6 days from setup to blastopore closure. This is consistent with research indicating that in fish with longer incubation times, such as brown trout (*Salmo trutta*) or rainbow trout (*Oncorhynchus mykiss*), the influence of SMF on the spatial orientation of embryos was greater than in fish with shorter incubation times, such as pike (Formicki et al. 1997; Tański 2002; Tanski et al. 2005). The significant impact of exposure time to EMF on the directional response of embryos is evident from studies where a very short (60 min) exposure to EMF (100 mT, 50 Hz) at the beginning of embryogenesis (cleavage) did not show a significant effect on the alignment of embryos (Tański 2002; Formicki et al. 2021).

Another argument indicating that a longer exposure time and/or increased EMF values could significantly affect the directional alignment of pike embryos is the experiment conducted on brown trout eggs incubated under the influence of EMFs generated by the energy infrastructure of a large power plant. In that case, with a species with embryogenesis significantly longer than that of pike and EMF values several times higher than under the HVTL, significant deviations from the natural axis of embryo alignment in GMF were observed (Krzystolik et al. 2024).

Our results also indicate increased mortality of pike eggs until blastopore closure incubated at the first research site under the 110 kV HVTL compared to the control group. However, at the site where eggs incubated under the influence of EMF from the 220 kV HVTL, such regularities were not observed, despite higher EMF values than at the first site. Therefore, it is assumed that the increased mortality was caused by factors other than EMF, e.g., the development of pathogenic microorganisms. However, it should be added that specific EMF values can also influence the development of potential pathogenic microorganisms, often leading to their inhibition (Mazurkiewicz-Zapałowicz et al. 2015; Korzelecka-Orkisz et al. 2016; Cordero-Samortin et al. 2020), although stimulating effects on their growth have also been reported (Kiełbasa et al. 2022).

The assumption that the increased mortality of pike eggs was most likely not caused by EMF is supported by previous laboratory studies on the effect of EMF and SMF on pike eggs (Fey et al. 2019a, b).

## Conclusion

From the obtained results, it can be concluded that the electromagnetic fields (EMF) originating from the high-voltage transmission lines (HVTL) did not exert a significant influence on the spatial arrangement of pike embryos and the survival of their eggs until blastopore closure. However, potential larger effects could be triggered with elevated EMF values or an extended exposure duration. These findings may necessitate reassessment in the future, particularly with the proliferation of more extensive power transmission networks.

## Data Availability

The data generated and analyzed during the current study are available from the corresponding author upon reasonable request.
